# Gas Uptake and Thermodynamics in Porous Liquids Elucidated by ^129^Xe NMR

**DOI:** 10.1021/acs.jpclett.4c00223

**Published:** 2024-05-09

**Authors:** Sarah E. Mailhiot, Petri Peuravaara, Benjamin D. Egleston, Rachel J. Kearsey, Jiří Mareš, Sanna Komulainen, Anne Selent, Anu M. Kantola, Andrew I. Cooper, Juha Vaara, Rebecca L. Greenaway, Perttu Lantto, Ville-Veikko Telkki

**Affiliations:** † NMR Research Unit, Faculty of Science, University of Oulu, P.O.Box 3000, FI-90014 Oulu, Finland; ‡ Department of Chemistry, Molecular Sciences Research Hub, Imperial College London, London, W12 0BZ, U.K.; ¶ Department of Chemistry and Materials Innovation Factory, University of Liverpool, Crown Street, Liverpool L69 7ZD, U.K.

## Abstract

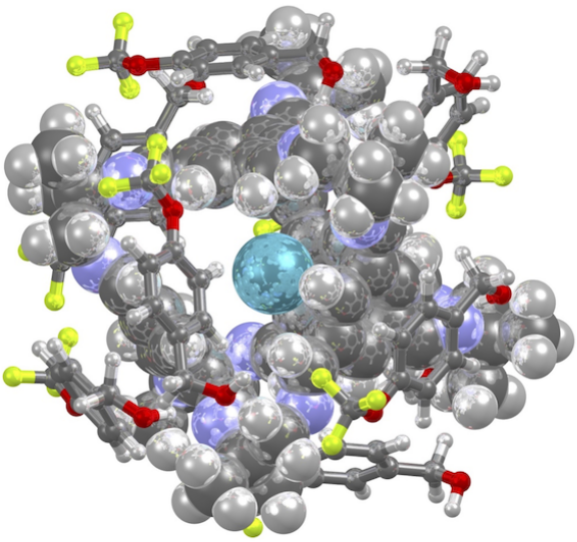

We exploited ^129^Xe NMR to investigate xenon gas uptake and dynamics in a porous liquid formed by dissolving porous organic cages in a cavity-excluded solvent. Quantitative ^129^Xe NMR shows that when the amount of xenon added to the sample is lower than the amount of cages present (subsaturation), the porous liquid absorbs almost all xenon atoms from the gas phase, with 30% of the cages occupied with a Xe atom. A simple two-site exchange model enables an estimate of the chemical shift of ^129^Xe in the cages, which is in good agreement with the value provided by first-principles modeling. *T*_2_ relaxation times allow the determination of the exchange rate of Xe between the solvent and cage sites as well as the activation energies of the exchange. The ^129^Xe NMR analysis also enables determination of the free energy of confinement, and it shows that Xe binding is predominantly enthalpy-driven.

Porous liquids (PLs) are liquids with permanent intrinsic cavities.^1^ PLs are promising materials for use in industrial-scale gas separation via continuous flow processes due to their potential for enhanced gas uptake, gas selectivity, and lower energy for regeneration compared to neat solvents and conventional liquid sorbents.^2^ One approach to forming a PL is to dissolve discrete porous molecules in a cavity-excluded solvent. For example, porous organic cages (POCs) containing permanent cavities, accessible through windows in the structure, can be dissolved in a range of different size-excluded solvents.^3−6^ To be size-excluded, the individual solvent molecules must be too large to pass through the window openings and occupy the POC cavities. In addition, the solvent must dissolve the POCs at a high enough concentration to achieve a reasonable pore volume and demonstrate an enhancement in gas uptake. One strategy to increase the solubility of POCs is dynamic covalent scrambling in which a statistical distribution of vertex-disordered POCs is formed using a mixture of two vicinal diamines.^5,7^ In the case of scrambled **CC3**^**3**^:**13**^**3**^–*R*, the POC mixture is prepared from the precursors for **CC13** and **CC3**–*R* to produce a binomial distribution of structurally related cages with cyclohexyl and dimethyl verticles (Figure 1b).^4^ These PLs have shown significantly enhanced uptake of CH_4_, CO_2_, Xe, N_2_, and SF_6_ compared to the neat solvents, with the gas uptake influenced by both the solvent and cage concentration.^4,5^ It is hypothesized that the overall gas uptake in a PL is dependent on gas (guest)-cage (host) interactions, gas binding in the cavity, the solubility of the gas in the solvent, the viscosity of the solvent and the resulting PL, and whether any peripheral cage functionalities or solvent molecules occupy the face openings and cavities of neighboring POCs.^5,8^

**Figure 1:**
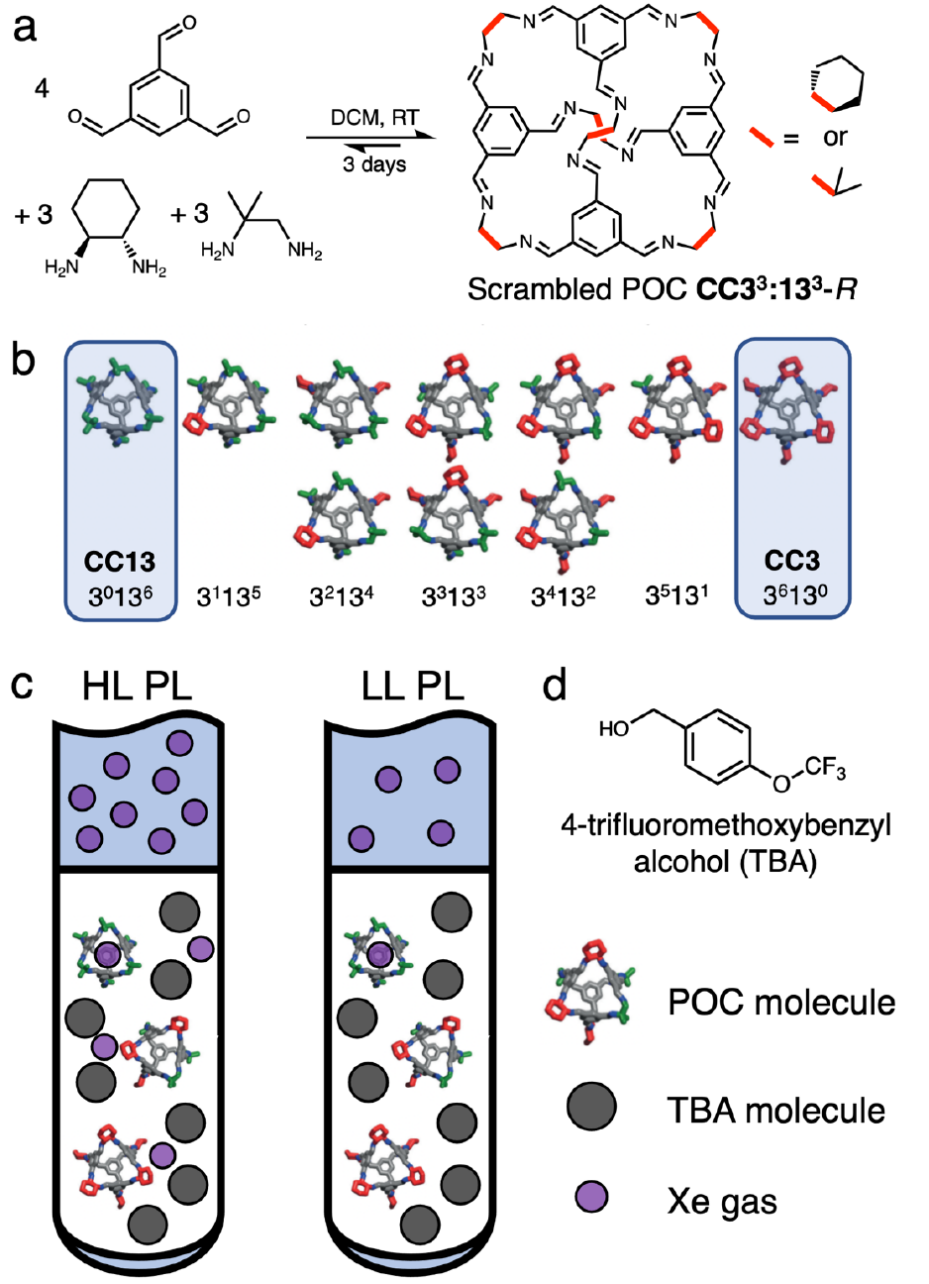
(a) Synthesis of a scrambled **CC3**^**3**^:**13**^**3**^–*R* cage mixture. (b) Chemical structures of the scrambled **CC3**^**3**^:**13**^**3**^–*R* mixture with either cyclohexane (red) or dimethyl (green) vertices, and hydrogens omitted for clarity. The parent cages **CC3**-*R* (**3**^**6**^**13**^**0**^) and **CC13** (**3**^**0**^**13**^**6**^) are highlighted. (c) Representative schematic showing porous liquid samples formed using 96 μmol of the scrambled porous organic cages (POCs) dissolved in cavity-excluded TBA (c, gray circles) and loaded with Xe gas (purple circles). The HL and LL PL samples contain 249 μmol and 56 μmol of Xe gas, respectively. The liquid volume at the bottom of sample tube is 0.41 mL and the gas volume at the top of the tube is 0.66 mL (total volume 1.07 mL).

Gaining an understanding of the gas uptake mechanism in PLs will enable the design and optimization of more effective PLs for gas capture and storage. One method for studying the key host–guest interactions between a gas and the pore cavities and solvent is ^129^Xe Nuclear Magnetic Resonance (NMR) spectroscopy. The ^129^Xe isotope has a nuclear spin of 1/2 and a relatively high gyromagnetic ratio. Furthermore, the ^129^Xe NMR chemical shift is highly sensitive to the atom’s local physical and chemical environment. For example, ^129^Xe NMR has been shown to be an excellent probe for various materials, which include both pure solvents^9,10^ as well as porous materials^11−22^ such as solid POCs,^23,24^ supramolecular cages,^25−27^ biosensors,^28−32^ metal–organic frameworks,^33−36^ molecular wheels,^37^ zeolites,^38^ ionic liquids,^39^ cements and shales,^40^ geopolymers,^41^ rare-earth element phosphates,^42^ clathrates,^43,44^ and fullerenes.^15,45,46^ Furthermore, it is possible to extract detailed information about the local environment of the ^129^Xe atom by combining experimental studies with quantum-chemical calculations and molecular simulations.^10,23,25,26,34,35,39,43,45−50^

When POCs pack together in the solid state, both the internal cavity and interstitial window sites can contribute to the gas adsorption process.^51^ Our previous work combined experimental and computational ^129^Xe NMR to reveal detailed information about ^129^Xe gas adsorption in solid **CC3**-*R*.^23,24^ The experimentally observed, exchange-averaged, and first-principles modeled ^129^Xe chemical shifts of ^129^Xe occupying the cage’s cavity and window sites allowed for determination of the occupancy and binding constants of ^129^Xe in **CC3** cavities, as well as the exchange rate of ^129^Xe between these sites.

In the present work, we combine, for the first time, experimental quantitative ^129^Xe NMR with quantum-chemical calculations to investigate xenon gas uptake and dynamics in PLs. Specifically, we focus on a PL formed by dissolving scrambled **CC3**^**3**^:**13**^**3**^–*R* in 4-(trifluoromethoxy)benzyl alcohol (TBA).^5^ TBA was chosen because it is one of the six novel size-excluded solvents with a high solubility of **CC3**^**3**^:**13**^**3**^. It is also significantly easier to source, handle and purify than other solvents.^4,5^ It is important to note that ^129^Xe NMR enables studying gas uptake in PL samples at the equilibrium, whereas in previous gas uptake and evolution studies on the same system, the PLs were first saturated by bubbling excess Xe gas through the solution over an extended time period.^5^

We prepared two PL samples including 96 μmol of cages in TBA, with the cage concentration of approximately 225 mM and the final liquid volume of approximately 0.42 mL. In addition, the sealed sample tubes included also either 249 μmol (equating to a Xe:cage molar ratio of 2.6, i.e., the Xe was in excess) or 56 μmol (equating to a Xe:cage molar ratio of 0.6, i.e., the cage was in excess) of ^129^Xe, denoted as high-loading (HL) and low-loading (LL) samples, respectively. The liquid volume at the bottom of the sample tube was about 40% of the total volume (Figure 1). For comparison to the PLs, two neat TBA samples were also prepared, corresponding to the HL and LL samples.

^129^Xe NMR spectra of the LL and HL (PL and TBA) samples are shown in Figure 2a and b. Both the PL and neat TBA solvent spectra include only a single ^129^Xe resonance. The peaks of xenon in the PL samples are broader and smaller than those of xenon in the neat solvent samples. Despite this lower height, the integrals of the LL PL sample are significantly larger than those of the LL TBA sample due to much greater width.

**Figure 2:**
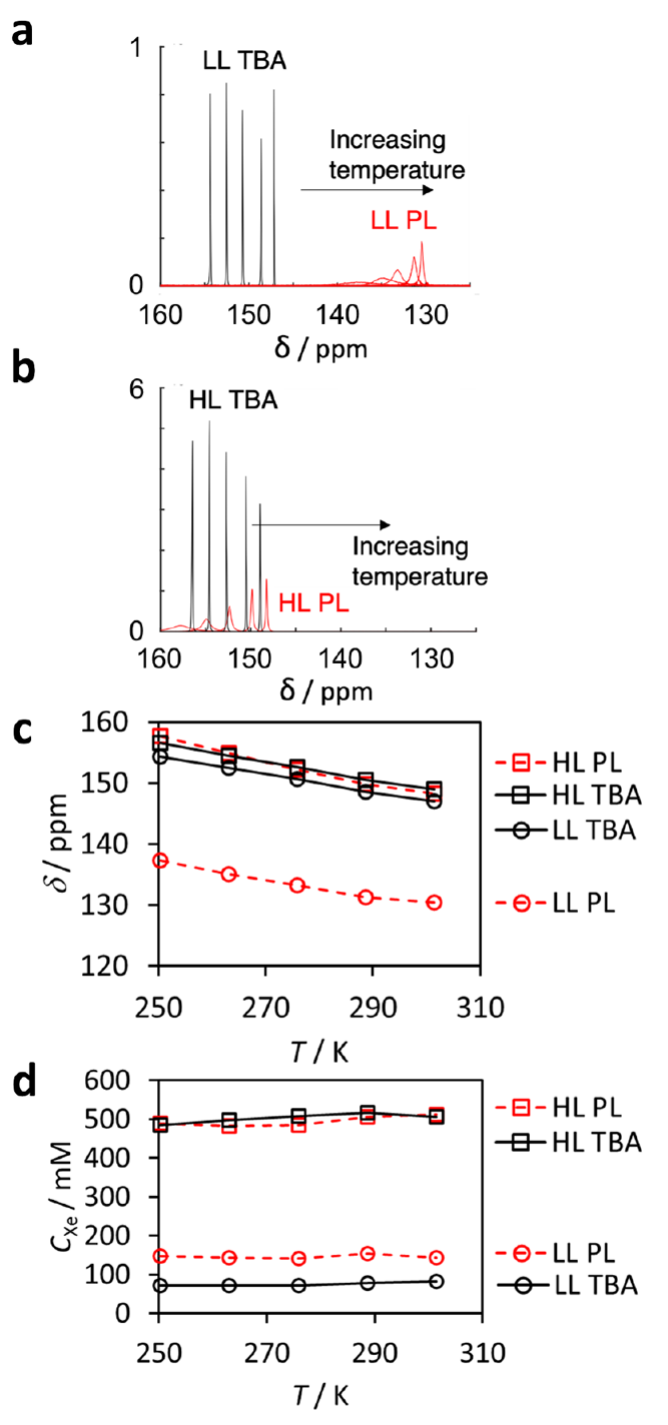
Variable-temperature (260–300 K) ^129^Xe NMR spectra of the (a) low-loading (LL) and (b) high-loading (HL) PL (red) and TBA solvent (black) samples. (c) ^129^Xe chemical shifts as a function of temperature. (d) The quantities of Xe dissolved in the samples as determined from ^129^Xe signal integrals.

Quantitative relaxation-corrected FID signals (see Materials and Methods) show that the Xe concentration in the LL PL sample ([Xe]_PL_ = 147 mM at 301 K) is about two times higher than that in the LL TBA sample ([Xe]_TBA_ = 82 mM at 301 K) due to the presence of accessible POC cavities in the PL (Figure 2d). The concentrations remain relatively constant over the whole measurement temperature range 250–301 K. The Xe concentration in the gas volume above the liquid was estimated to be close to zero in the LL PL sample, indicating that the PL absorbed almost all Xe atoms from the gas phase. In contrast, the Xe concentration in the gas volume in the TBA sample was estimated to be approximately 21 mM at 301 K, corresponding to 0.52 atm pressure.

If we assume that the concentration of xenon in the solvent of the LL PL sample is equal to that in the LL TBA sample (82 mM at 301 K), then the remaining Xe atoms (147 mM - 82 mM = 65 mM) have to be inside the cages; i.e., about 56% of the Xe atoms are located in the bulk solvent and 44% are located within the cages. As the concentration of cages is 234 mM, this equates to 30% occupancy of the cages in the LL PL by Xe atoms. This approximation does not take into account the decreased partial volume of solvent in the PL after the addition of cages, and the effect of different gas pressures in the gas volume on Xe solubility. However, the two-site exchange analysis described below indicates that the solvent and cage site fractions obtained by this approximation are reasonable.

In the HL samples, the Xe concentrations in the PL and TBA solvent samples are almost equal (513 and 506 mM at 301 K) - this is likely because there are more Xe atoms than cages (225 mM) in the PL sample, meaning that the base solubility of Xe in the TBA solvent predominantly dictates the uptake in both. Xe concentrations in the gas volumes were estimated to be 51 and 47 mM, corresponding to 1.25 and 1.16 atm pressures.

The observation of a single ^129^Xe resonance implies that either Xe occupies a single environment, or it is in rapid dynamic exchange between different environments on the NMR time scale.^52^ When POCs are packed together in the solid state, Xe atoms occupy two different environments with significantly different ^129^Xe chemical shifts;^23^ they can be encapsulated in the intrinsic cage cavities (only one Xe fits in a cage), or occupy the window cavity formed between two neighboring cages. In this PL, discrete POCs are fully dissolved in the size-excluded solvent, meaning that only the internal cavities remain, and the window cavities are no longer present. However, unlike in solid POCs, the solvent itself needs to be taken into account as an environment, since Xe can be dissolved in TBA. Taking all of this into account, we hypothesize that a two-site model, in which Xe can either be found to occupy the intrinsic cage cavities or located in the bulk solvent, is a good approximative model for this PL.

In this model, the observed ^129^Xe chemical shift of xenon in the PL is the population-weighted average of the chemical shifts of Xe in the cage (δ_cage_) and solvent (δ_sol_):^52^
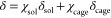
1Here, χ_sol_ = [Xe]_TBA_/[Xe]_PL_ and χ_cage_ = 1 – χ_sol_ are the relative amounts of Xe in the solvent and cages, respectively. As the chemical shift of Xe in the solvent is known from the LL TBA spectra (δ_sol_ = 147 ppm at 301 K) and the chemical shift of Xe in the LL PL sample is δ = 130 ppm at 301 K, it is possible to estimate the chemical shift of the cage site by eq 1, using χ_cage_ = 0.44 obtained from the quantitative ^129^Xe NMR signal analysis. The resulting value is 110 ppm (37 ppm less than the chemical shift of xenon in the pure solvent) at 301 K.

The two-site exchange analysis was not applied to the HL PL sample because there is very little difference in the chemical shift or amount of dissolved Xe between the HL PL and neat TBA sample, indicating that most of the Xe is located in the solvent. In the HL and LL samples, the chemical shift decreases with increasing temperature both in the PL and TBA solvent samples (Figure 2a–c), most probably predominantly due to decreasing solvent density.^10,53^

We note that, in a recent paper, a multiple-site exchange model was proposed to explain the ^129^Xe chemical shifts in a porous liquid including Noria-OEt hosts dissolved in 15-crown-5 solvent.^54^ However, this porous liquid is more complex than the PL studied in the present article, for example, due to the potential formation of Xe-crown ether complexes. The experimental observations, supported by the computational simulations described below, indicate that the two-site exchange model is a reasonably good approximation for the PL studied here.

To check the validity of the two-site exchange model for the PL, we performed quantum-chemical modeling using the Turbomole^55^ code at density functional theory (DFT) level including scalar relativistic (X2C) effects^56^ for the ^129^Xe chemical shift both within the cages and the solvent. The computational details are described in the SI. Best chemical-shift estimates were obtained with the hybrid BHandHLYP DFT functional.^57−59^ It has previously been shown to provide the best estimates among a variety of DFT functionals for the Xe chemical shifts in different environments^10,23,25,26,43,45^ and was found to be essential for the correct chemical shift difference between the PL cages and the neat TBA solvent. While the correct sign was already obtained for the shift difference at the lower x2c-SVPall^60^ basis-set level analyzed for the surrounding atoms, much improved quantitative agreement with experimental values (see below) required adding an x2c-TZVPall^60^ basis-set correction carried out for each snapshot using the efficient but less accurate PBE functional.^61^ In all calculations, x2c-TZVPall-s basis set optimized for NMR shielding was used for Xe atom.^62^

Since the scrambled **CC3**^**3**^:**13**^**3**^–*R* includes a mixture of cages with different functionalization on the cage periphery, the average ^129^Xe shift inside these cages was modeled as the average of the two values for the two corresponding parent cages, **CC3**-*R* and **CC13**, i.e., the two cage species that are structurally the most different in the mixture (Figure 1). Molecular dynamics (MD) simulations were carried out with the semiempirical GFN2-xTB method^63^ using the xTB code^64^ for a cage (either **CC3**-*R* or **CC13**) in a droplet of 164 explicit TBA solvent molecules.

Despite the difference in periphery functionalization, the calculated time-averaged Xe chemical shifts (wrt atomic xenon) at *T* = 300 K are very similar, 104 ± 7 ppm and 94 ± 6 ppm for **CC3**-*R* and **CC13**, respectively, which gives an average 99 ± 10 ppm computational shift for δ_cage_. This is in relatively good agreement with the shift of the cage site given by the experiments and the exchange model (110 ppm). The simulated chemical shift of Xe in the neat TBA solvent, 143 ± 6 ppm, is also in good agreement with the experimental value (147 ppm). Consequently, the simulated ^129^Xe chemical shift difference between the solvent and cage, 44 ± 11 ppm, is in good agreement with the experimentally observed difference of 37 ppm. Besides a droplet approximation being used with a relatively small number of TBA molecules, the present simulations also entail the approximations inherent to the GFN2-xTB method. Despite this, both the shift difference and the absolute ^129^Xe chemical shifts in the two different environments are well reproduced. Overall, the modeling supports that the two-site exchange model is a reasonably good approximation for the current system; i.e., it indicates that contributions from other potential sites can be ignored as their effect on the system is relatively small.

In the PL samples, there is an equilibrium of Xe within three different pools. The Xe dissolved in the PL is in equilibrium with the Xe in the gas phase above the liquid. Additionally, there is an equilibrium of Xe between the cage cavity and bulk solvent in the PL. The reaction equation describing the equilibrium state within the PL is
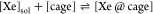
2Here, [Xe]_sol_ and [Xe@cage] are the concentrations of Xe in the solvent and cages, respectively, as determined above, and [cage] is the known concentration of the cages (234 mM for the LL PL sample). Therefore, the equilibrium constant is *K*_eq_ = [Xe@cage]/([Xe]_sol_[cage]). The Gibbs free energy of reaction, *RT* ln *K*_eq_, can be calculated from the equilibrium constant as Δ*G* = −1.61 ± 0.15 kJ mol^–1^ at 301 K. Fit of Δ*G* = Δ*H* – *T*Δ*S* with the calculated Gibbs free energies as a function of temperature (Figure 3a) resulted in a change of enthalpy of Δ*H* = −1.6 ± 0.4 kJ mol^–1^ and entropy of Δ*S* = 0.1 ± 1.3 J mol^–1^ K^–1^ upon encapsulation. This indicates that Xe binding is predominantly enthalpy-driven. Dissolution in conventional liquids requires energy for forming a cavity for the solute. However, in the case of PLs, the total volume taken by the solute and, hence, enthalpy decreases as xenon enters to the permanently accessible cavities.^65^

**Figure 3:**
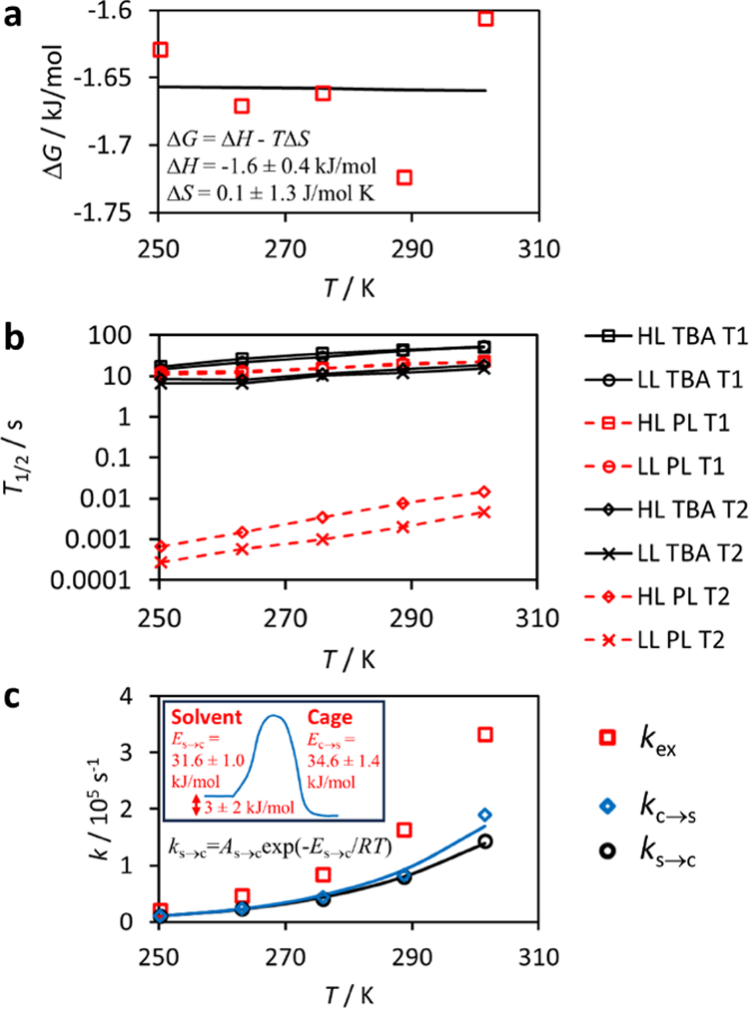
(a) Gibbs free energy of the reaction, in which a Xe atom in a solvent pool and a cage form a complex of Xe encapsulated in the cage, as a function of temperature, and the fit of Van’t Hoff equation (solid line). (b) *T*_1_ and *T*_2_ relaxation times of ^129^Xe in the LL and HL PL, as well as neat TBA solvent samples as a function of temperature. (c) Exchange rates of Xe moving from cage to solvent (*k*_c→s_) and solvent to cage (*k*_s→c_), and their sum (*k*_ex_). The fits of the Arrhenius equation with the experimental data are shown by solid lines. Inset: visualization of the energy barrier for xenon encapsulation.

The dynamics of xenon in the PL and solvent samples was further investigated by relaxation measurements (Figure 3b). In the neat TBA samples, the *T*_1_ relaxation time of ^129^Xe increases from 15 to 52 s as the temperature increases from 250 to 301 K. In the PL samples, the *T*_1_ relaxation times are slightly shorter, varying from 12 to 23 s over the same temperature range. The *T*_1_ values are quite independent of Xe loading.

However, the *T*_2_ values of the TBA samples (7–19 s) are many orders of magnitude longer than those of the PL samples (0.3–14 ms). At 301 K, the *T*_2_ relaxation times of the LL and HL TBA solvent samples are 15 and 19 s, respectively, while they are 5 and 15 ms in the corresponding PL samples.

According to relaxation modeling,^24^ fluctuations in the isotropic chemical shift are known to be the dominating *T*_2_ relaxation mechanism for ^129^Xe in this kind of porous system, due to the large chemical shift difference between the exchanging sites. According to a two-site exchange model, the *R*_2_ relaxation rate, *R*_2_ = 1/*T*_2_, is inversely related to the exchange rate such that^66,67^
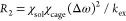
3where Δω = ω_sol_ – ω_cage_ is the resonance frequency difference between the solvent and cage sites, and *k*_ex_ = *k*_c→s_ + *k*_s→c_, where *k*_c→s_ and *k*_s→c_ are the exchange rates from cage to solvent and solvent to cage, respectively. The experimentally observed relaxation rate is assumed to be independent of the CPMG echo time due to fast exchange. This was experimentally confirmed at higher temperatures; however, experimental confirmation at the lowest temperature (250 K) was not possible due to very short *T*_2_. Figure 3c illustrates the exchange rates in the LL PL sample as calculated by eq 3 using the experimentally measured *R*_2_, as well as the aforementioned populations and chemical shifts. At 301 K, the exchange rate *k*_ex_ is 3 × 10^5^ s^–1^ and the corresponding exchange time, *k*_ex_^–1^, is 3 μs.

The temperature dependence of the exchange rate was modeled by the Arrhenius equation
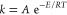
4Here, *E* is the exchange activation energy and *R* the universal gas constant. Fit of the Arrhenius equation with the experimentally observed exchange rates (Figure 3c) results in *E*_s→c_ = 31.6 ± 1.0 kJ mol^–1^ and *E*_c→*s*_ = 34.6 ± 1.4 kJ mol^–1^. These values reflect the height of the energy barrier for xenon encapsulation as visualized in the inset of Figure 3c. The energy difference *E*_s→c_ – *E*_c→s_ = 3 ± 2 kJ mol^–1^ gives an estimation of how much the energy of the system is decreased in the encapsulation process. Within the error bars, the value is in good agreement with the change of enthalpy (Δ*H* = −1.6 ± 0.4 kJ mol^–1^) obtained by the Van’t Hoff analysis.

In conclusion, we demonstrated that combined experimental and computational ^129^Xe NMR analysis provides versatile thermodynamic information about Xe uptake in porous liquids (PL). Specifically, we studied Xe uptake in a porous liquid consisting of scrambled porous organic cages dissolved in 4-(trifluoromethoxy)benzyl alcohol (TBA). ^129^Xe signal amplitudes showed that in equilibrium the low-loading PL sample absorbs almost all xenon atoms from the gas phase. A simple two-site exchange model enabled estimation of the chemical shift of Xe encapsulated in the cage cavities, as well as evaluation of the amounts of Xe adsorbed into the cages and dissolved in the solvent. It was found that approximately 44% of Xe atoms was inside cages and 56% in the solvent, and 30% of the cages was occupied by a Xe atom. Computational chemical shifts of the cage and solvent sites are in excellent agreement with the experimental values, supporting the validity of the two-site exchange model. We determined the equilibrium constants of Xe between the solvent and cage sites and concluded that the spontaneous Xe uptake into cages is predominantly enthalpy-driven. Based on the *T*_2_ relaxation data, the exchange rate between cage and solvent sites was estimated to be 3 × 10^5^ s^–1^ at 301 K, and the activation energy of the exchange is approximately 33 kJ mol^–1^. The information and methodology presented here can be used for further design of porous liquids, the pairing of porous organic cages with size-excluded solvents, and in evaluating the differences in the performance for porous liquids and solids.

## Materials and Methods

Synthesis of scrambled **CC3**^**3**^:**13**^**3**^–*R* and purification of the TBA solvent are described in the SI.^5,7^ Two PL samples were prepared using 100 mg (average MW = 1039, 96 μmol) of desolvated scrambled **CC3**^**3**^:**13**^**3**^–*R* cages in TBA so that the final liquid volume was about 0.41 and 0.43 mL in the LL and HL samples (see definitions of the abbreviations below). The concentrations of the cages were 234 and 225 mM, respectively. These PL samples were preloaded into medium-wall 5 mm NMR tubes (inner diameter 3.2 mm), prior to the introduction of differing amounts of ^129^Xe isotope-enriched (91.1%) xenon gas - either 249 μmol (equating to a Xe:cage molar ratio of 2.6, *i.e*., the Xe was in excess) or 56 μmol (equating to a Xe:cage molar ratio of 0.6, i.e., the cage was in excess) of ^129^Xe was added, denoted as high-loading (HL) and low-loading (LL) samples, respectively (Figure 1). For comparison to the PLs, two neat TBA samples (0.42 and 0.47 mL) were also prepared and loaded with 242 and 53 μmol of Xe, corresponding to the HL and LL samples. ^129^Xe was condensed into the sample tube using liquid nitrogen, and thereafter the sample was sealed with a flame. The inner volume of the sealed LL PL, LL TBA, HL PL and HL TBA samples was 1.07, 1.13, 1.04, and 1.06 mL, including 0.41, 0.47, 0.43, and 0.42 mL liquid volume at the bottom and 0.66, 0.66, 0.61, and 0.64 mL gas volume at the top (Figure 1), respectively. After the sealed sample was brought to room temperature, the sample was left to equilibrate so that the Xe was distributed between the liquid and gas volumes. A reference sample including 219 mM ^129^Xe isotope-enriched (91.1%) xenon gas (5.3 atm pressure at room temperature) was used to convert the signal intensities to the molar ratio of xenon.

^129^Xe NMR measurements were performed on a Bruker Avance III 600 (14.1 T) spectrometer with a 5 mm broad-band (BBO) probe. ^129^Xe resonance frequency was 166 MHz. The spectra were measured with a sweep width of 300 ppm, an acquisition time of 1 s, and 64 scans. The relaxation delay was 25 s for the PL samples, 50 s for the TBA samples, and 1.5 h for the gas reference sample. Only one scan was used for the gas reference sample. The experiment times were 28 min, 55 min, and 1.5 h for the PL, TBA, and gas reference samples, respectively.

*T*_2_ relaxation data was measured using the Carr–Purcell–Meiboom–Gill (CPMG) sequence.^68^ For the PL samples, the echo time was 0.1 ms and 1 to 100 echoes (total echo time 0.1 to 10 ms) were used to detect the relaxation decay with 11 log-spaced steps. The relaxation delay was 25 s, and 24 scans were recorded within the experiment time of 1 h 51 min. For the TBA samples, the echo time was 4 ms, and 4 to 5000 echoes (total echo time 0.016 to 20 s) were used to detect the relaxation decay with 11 log-spaced steps. The relaxation delay was 50 and 24 scans were recorded with the experiment time of 4 h 45 m.

*T*_1_ relaxation data was measured using the inversion–recovery sequence. The recovery times varied between 0.01 and 30 s, the number of steps was 9–15, the number of scans was 32 and the relaxation delay was 30 s.

^129^Xe NMR spectra and *T*_2_ relaxation data were collected from 250.3 to 301.5 at 12.8 K intervals with a temperature stabilization delay of 30 min.

Quantitative ^129^Xe NMR signal analysis was done based on the intensity of the free induction decay (FID) signal. The decay was very fast in the case of the PL samples, especially at the lowest temperatures, and the signal decayed significantly during the dead time, which is the time between the center of the excitation pulse and the detection of the first point. Therefore, FID intensities extrapolated to zero time were used in the quantitative analysis (0–20% correction). The magnetization was not fully relaxed to equilibrium before the repetitions of the experiments, because the repetition time *T*_*R*_ including the relaxation delay and acquisition time (26 s for the PL samples and 51 s for the TBA samples) was relatively short as compared to *T*_1_ relaxation times (12–23 s for the PL samples and 15–52 s for the TBA samples). This was compensated for by dividing the intensity by the factor of 1 – exp(−*T*_*R*_/*T*_1_) (3–60% correction). The corrected, quantitative signal intensities were converted into concentrations by comparing them to the signal intensity of the reference gas sample with a 219 mM xenon concentration.

## Supporting Information Available

Additional details about synthesis and purification of materials as well as computational methods and modeling, including table of results and figures of model systems as well as statistical convergence and thermal averages of ^129^Xe NMR chemical shifts (PDF)
