# Supplementary material for: Gas Uptake and Thermodynamics in Porous Liquids Elucidated by 129Xe NMR
Source: J Phys Chem Lett. 2024 May 9;15(20):5323–30. doi: 10.1021/acs.jpclett.4c00223 (PMC11129303; doi:10.1021/acs.jpclett.4c00223)
Supplement: Supplementary file 1 — jz4c00223_si_001.pdf [file jz4c00223_si_001.pdf]

Electronic supplementary information (ESI) for

Gas Uptake and Thermodynamics in Porous Liquids Elucidated by ^{129}Xe NMR

Sarah E. Mailhiot,[†] Petri Peuravaara,[†] Benjamin D. Egleston,[‡] Rachel J. Kearsey,[¶] Jiří Mareš,[†] Sanna Komulainen,[†] Anne Selent,[†] Anu M. Kantola,[†] Andrew I. Cooper,[¶] Juha Vaara,[†] Rebecca L. Greenaway,[‡] Perttu Lantto,[*,†] and Ville-Veikko Telkki[*,†]

†*NMR Research Unit, Faculty of Science, University of Oulu, P.O.Box 3000, FI-90014 Oulu, Finland*

‡*Department of Chemistry, Molecular Sciences Research Hub, Imperial College London, London, W12 0BZ, UK*

¶*Department of Chemistry and Materials Innovation Factory, University of Liverpool, Crown Street, Liverpool L69 7ZD, UK*

E-mail: perttu.lantto@oulu.fi; ville-veikko.telkki@oulu.fi

Contents

1 Materials **S2**

1.1 Synthesis of scrambled **CC3^3:13^3**$-R$ S2

1.2 Purification of 4-(trifluoromethoxy)benzyl alcohol (TBA) S3

2 Computational details **S4**

2.1 Molecular dynamics simulations S4

2.2 Quantum-chemical calculations S7

2.2.1 Spin-orbit effect on the Xe shift S11

References **S13**

1 Materials

1.1 Synthesis of scrambled CC3^3:13^3$-R$

1,3,5-Triformylbenzene was purchased from Manchester Organics (UK), *R,R*-cyclohexyl-1,2-diamine and 1,2-diamino-2-methylpropane were purchased from TCI (UK), 4-(trifluoromethoxy)benzyl alcohol was purchased from Fluorochem (UK) and dichloromethane was purchased from VWR.

Two batches carried out using the following procedure: (*R*,*R*)-Cyclohexyl-1,2-diamine (1.637 g, 14.3 mmol, 3 eq.), 1,2-diamino-2-methylpropane (1.264 g, 14.3 mmol, 3 eq.), and 1,3,5-triformylbenzene (TFB, 3.099 g, 19.1 mmol, 4 eq.) were dissolved in dichloromethane (DCM, 1.35 L). The reaction mixtures were stirred at room temperature for 3 days. The solutions were filtered to remove insoluble precipitate before both batches were combined and the DCM was removed by rotary evaporation. The resulting crude cream solid was washed with ethyl acetate (3 $\times$ 150 mL) before being re-dissolved in the minimum amount of DCM (ca. 35 mL), filtered, and recovered by rotary evaporation, to afford **CC3^3:13^3**$-R$

as a cream solid which was dried in a vacuum oven at 90 °C overnight (7.416 g, 7.13 mmol, 72%).

^{1}**H NMR** (400 MHz, $CDCl_3$) δ_H (ppm) 8.17–7.81 (24H, m, N=CH and ArH), 3.94–3.35 (12H, m, CHN=CH), 1.91–1.32 (42H, m, CH_2 and CH_3); **HRMS** (ESI+) calc. for scrambled cages $3^0 13^6$ $C_{60}H_{72}N_{12}$ 960.6003, $3^1 13^5$ $C_{62}H_{74}N_{12}$ 986.6159, $3^2 13^4$ $C_{64}H_{76}N_{12}$ 1012.6316, $3^3 13^3$ $C_{66}H_{78}N_{12}$ 1038.6472, $3^4 13^2$ $C_{68}H_{80}N_{12}$ 1064.6629, $3^5 13^1$ $C_{70}H_{82}N_{12}$ 1090.6785, $3^6 13^0$ $C_{72}H_{84}N_{12}$ 1116.6942; found $[M+H]^+$ 961.5754, 987.5898, 1013.6079, 1039.6222, 1065.6359, 1091.6484. Data in accordance with literature values[1].

1.2 Purification of 4-(trifluoromethoxy)benzyl alcohol (TBA)

Purification of the solvent was performed by vacuum distillation - first and last 10% of distillate was disposed of, and TBA was collected at steady state (72 °C @ 1.9 x 10^{-1} mbar, brown-red solution to near colourless liquid).

^{1}**H NMR** (400 MHz, $CDCl_3$) δ_H (ppm) 7.28 (d, J = 8.7 Hz, 2H), 7.16 (d, J = 8.0 Hz, 2H), 4.54 (s, 2H), 3.73 (s, 1H); ^{13}**C NMR** (101 MHz, $CDCl_3$) δ_C (ppm) 148.7, 139.5, 128.3, 121.1, 120.6 (q, J = 256.9 Hz), 64.0. Data in accordance with literature values [2].

2 Computational details

2.1 Molecular dynamics simulations

Molecular dynamics (MD) simulations for the studied systems were run with the xTB program package[3]. Separate simulations for both the **CC3**-R and the **CC13** cage structures were carried out and in both cases a xenon atom was placed inside the PL cage that, in turn, was surrounded by 164 TBA solvent molecules. In addition, simulations were performed for a xenon atom surrounded by only the 164 TBA molecules. The semi-empirical, extended tight-binding method GFN2-xTB[4] was used to generate the energy and forces for the leap-frog MD algorithm run with a 1 fs timestep in all the three cases. The simulations were carried out in the constant particle number, volume and temperature (NVT) ensemble with the thermostat set to a constant temperature of 300 K.

The initial geometries for the MD simulations, depicted in Figure S1, were determined using the Packmol program[6]. For **CC3**-R and the **CC13** simulations, a cage molecule with a xenon atom inside was placed at the centre and the TBA molecules were placed evenly around it within a radius of 25.0 Å. For the simulation without the cage, the TBA molecules were placed around a xenon atom at the centre, again within a 25.0 Å radius. These geometries were then optimized by using the xTB program package with the same GFN2-xTB method that was also used in the following MD simulations.

In the MD simulations, a logfermi type spherical cavity potential of $R_{sphere} = 40\,a_0$ (21.2 Å) radius was used to keep the solvent molecules confined around the origin. The cages with encapsulated Xe atom were further confined at the center of solvent with the cavity potential of the same type, with $R_{\mathrm{sphere}} = 25\,a_0$ (13.2 Å). In the TBA simulations without the cage molecule, the Xe atom was confined at the center of the solvent using smaller cavity potential with $R_{\mathrm{sphere}} = 15\,a_0$ (7.9 Å).

In addition to the original simulation starting from the optimized structure, three and two independent MD simulations for **CC3**-R and the **CC13** systems, respectively, were

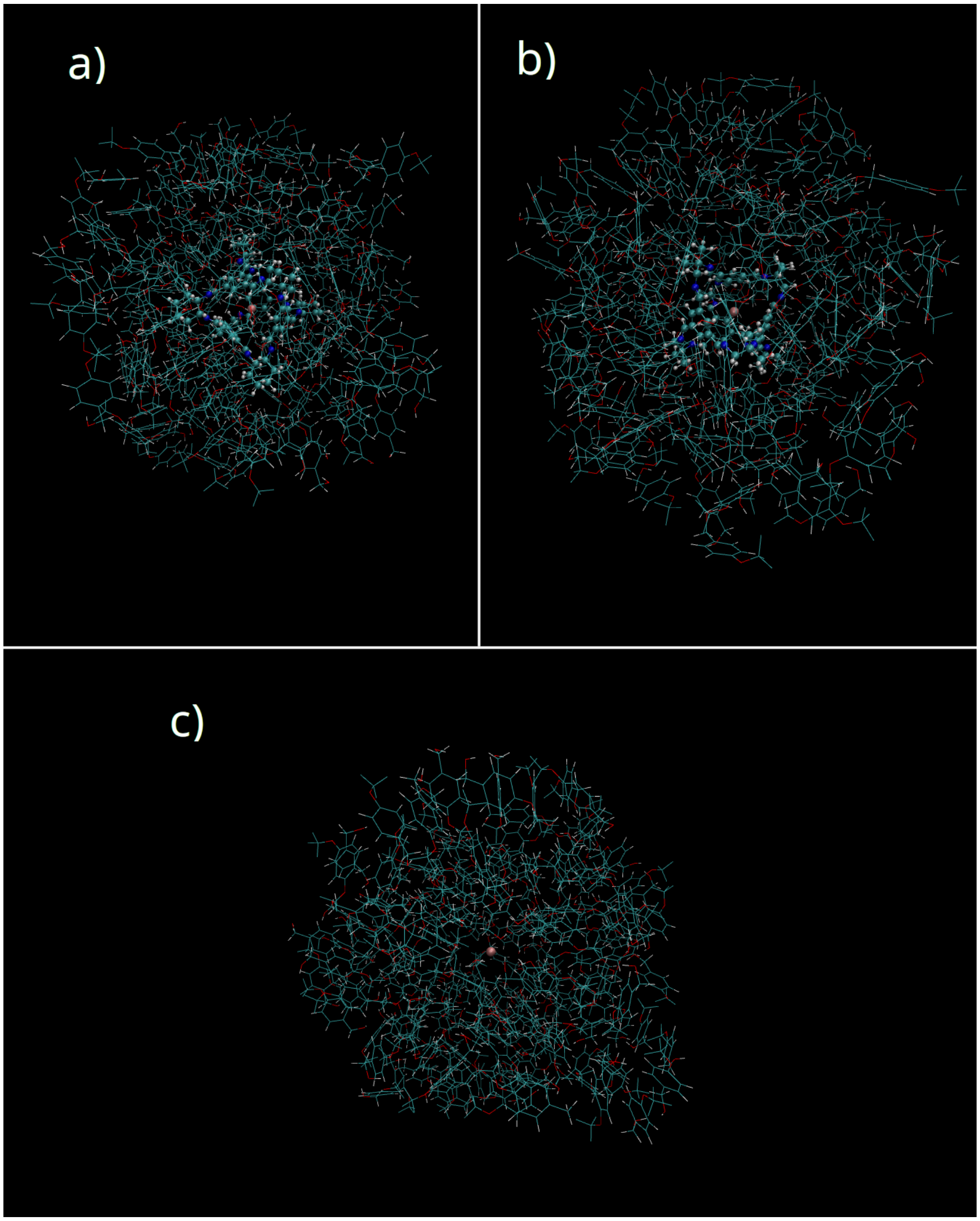

Figure S1. Starting geometries of the MD simulations for: a) **CC3-**R, b) **CC13**, and c) cageless, neat TBA structure. VMD program[5] was used for the graphics.

started from the same structure taken from the original simulation but with different starting velocities for the nuclei. The approach enabled trivial parallelism that provided more data and more efficient probing of the phase space.

A similar approach for the neat TBA system was not successful as the Xe gained additional velocity probably due to the interaction with the confinement potential in the begin-

ning (in other two systems only the cage is more strictly confined and Xe atom is moving freely inside the larger droplet confinement) resulting simulations drifting far from equilibrium. Therefore, only the original, well equilibrated TBA simulation was included in the averaging.

After the equilibration period of 5 ps, the snapshots were taken at 0.25 ps intervals from the simulation trajectories and Xe NMR shieldings were computed for the extracted cluster models. The data was collected from each simulation and combined together. Hence, 60 ps (one simulation), 186 ps (four), and 135 ps (three) of data for TBA, **CC3**-*R*, and **CC13**, respectively, were obtained for statistical analysis. The correlation length was estimated to be ≤ 1 ps with both data-halving[7] and block-averaging methods[8]. We chose a 1 ps sampling frequency for thermal averages and errors reported in Table S1. Convergence of the Xe chemical shifts is shown in Fig. S2 and thermal averages are displayed in Fig. S3.

2.2 Quantum-chemical calculations

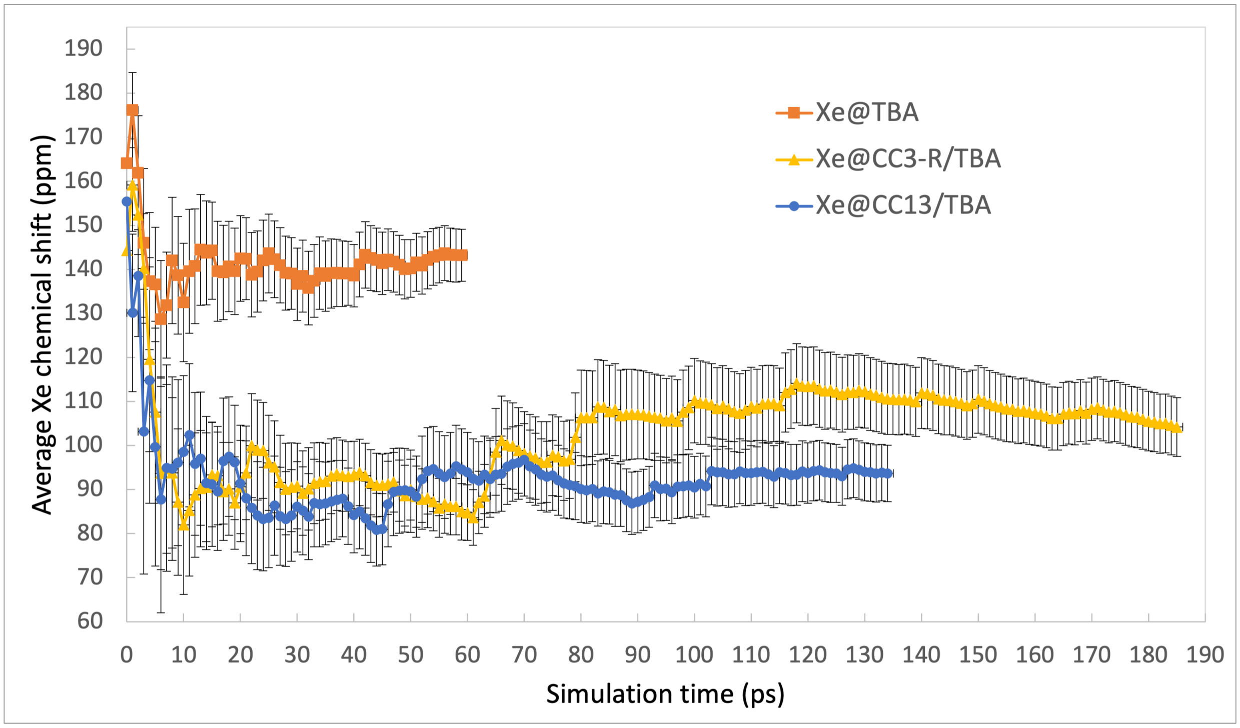

Figure S2. Convergence of the simulated Xe chemical shift as a function of the simulation time, *i.e.*, the number of snapshots with 1 ps sampling interval.

Thermally averaged ^{129}Xe NMR chemical shifts were obtained by carrying out quantum-chemical (QC) calculations for snapshots extracted from the MD simulation trajectories of three environments: the neat TBA solvent as well as inside the **CC3**-*R* or **CC13** cages in an explicit TBA solvent.

Each snapshot consisted of Xe atom, **CC3**-*R* or **CC13** cage when present, and 20 TBA molecules closest to the Xe atom. The fixed number or solvent molecules greatly simplified automatizing QC calculations for hundreds of snapshots and the number covers the first TBA solvation shell in each system. This was determined from the radial distribution functions (RDF) that give 18, 14 or 11 TBA molecules on average in the first solvation shell around the Xe atom in **CC3**-*R*, **CC13**, and neat TBA solvent, respectively.

^{129}Xe NMR shielding calculations with the Turbomole[9,10] code were carried out at scalar-relativistic X2C level[11] with x2c-TZVPall-s basis set for the Xe atom optimized for shielding

Table S1. Xenon chemical shift (ppm) simulation averages at T = 300 K ($\delta^{300\,\mathrm{K}}_{\mathrm{Xe}}$ w.r.t. $\sigma_{\mathbf{Xe\ atom}}$ = 5847.8 ppm) with standard errors of the mean (±SEM). X2C scalar-relativistic Xe NMR shielding calculations with different DFT functionals and basis sets are computed in the Turbomole code for the same 186, 135, and 60 snapshots of CC3-R, CC13, and neat TBA solvent, respectively.

	**CC3**-R	**CC13**	TBA[a]	PL[b]	(TBA - PL)[c]
P-SVP[d]	177 ± 8	171 ± 8	148 ± 7	174 ± 11	-26 ± 13
P-TZVP[e]	166 ± 8	158 ± 8	168 ± 7	162 ± 11	6 ± 13
B-SVP[f]	115 ± 6	107 ± 6	123 ± 6	111 ± 9	12 ± 11
B-TZVP[g]	104 ± 7	94 ± 6	143 ± 6	99 ± 9	44 ± 11
EXP	–[h]	–[h]	148	101	47
BSE[i]	-10.8 ± 0.5	-12.9 ± 0.3	19.9 ± 0.8		
DFE[j]	-62.4 ± 1.4	-64.6 ± 1.3	-24.6 ± 1.0		

[a] Xe chemical shift in neat TBA solvent.
[b] Estimated value for the scrambled $\mathbf{CC3}^3$:$\mathbf{13}^3$-R porous liquid (PL) is averaged over two independent averages for **CC3**-R and **CC13**: $\overline{Z} = 0.5(\overline{X} + \overline{Y})$. Linear propagation is used for the error estimate: $\mathrm{SEM}(\overline{Z}) = \sqrt{\mathrm{SEM}^2(\overline{X}) + \mathrm{SEM}^2(\overline{Y})}$.
[c] Xe chemical shift difference between Xe in PL cages and in neat TBA solvent. Linear propagation of error is used for the sum of estimates of independent variables, see footnote b.
[d] PBE calculations with x2c-TZVPall-s/x2c-SVPall basis sets for Xe/other atoms.
[e] PBE calculations with x2c-TZVPall-s/x2c-TZVPall basis sets for Xe/other atoms.
[f] BHandHLYP calculations with x2c-TZVPall-s/x2c-SVPall basis sets for Xe/other atoms.
[g] Best result obtained as an average over B-SVP + BSE result for each individual snapshots. [h] A single value for the scrambled $\mathbf{CC3}^3$:$\mathbf{13}^3$–R was obtained.
[i] Average basis set effect (BSE) (P-TZVP – P-SVP).
[j] Average DFT functional effect (DFE) (B-SVP – P-SVP).

calculations[12], as well as both x2c-SVPall and x2c-TZVPall basis sets[13] for other atoms. In order to efficiently carry out hundreds of preliminary NMR calculations, the pure PBE density functional[14] was used. It is expected to provide good estimates for the correction due to better basis set for other than Xe atom (x2c-TZVPall - x2c-SVPall), so-called basis set effect (BSE) listed in Table S1. It should also give reasonable first estimates for Xe shift differences in different environments.

However, due to the known overestimation of absolute Xe chemical shifts (w.r.t. free Xe atom in low-density gas) by the PBE functional, seen both in molecules[15–18] and material cavities[19–22], chemical shift estimates were improved by using the best-performing

[15–22] hybrid BHandHLYP functional[23–25]. Since BHandHLYP calculations are much more resource-consuming, they were only carried out with smaller x2c-TZVPall-s/x2c-SVPall basis set combination for Xe/other atoms. The best estimates for the Xe chemical shifts (B-TZVP in Table S1 and BHandHLYP/TZVP in Figure S3) were obtained by adding the above-mentioned BSE computed at the PBE level to the BHandHLYP value obtained with the smaller basis set in each uncorrelated snapshot, before averaging.

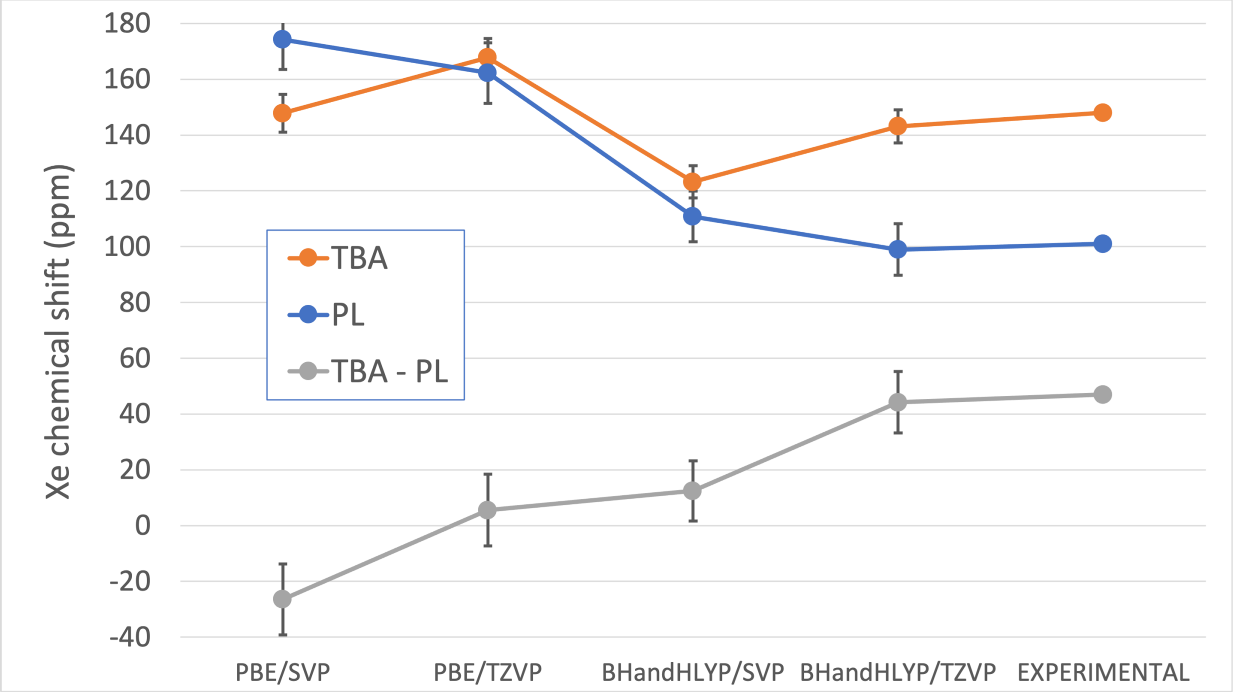

Figure S3. Thermal averages (T = 300 K) of ^{129}Xe chemical shits in porous liquid (PL) and in neat TBA solvent, as well as the shift differences with various DFT functional/basis set combinations for scalar-relativistic X2C NMR shielding calculations (see Table S1). Experimental results are also shown.

The best Xe chemical shift estimates at the scalar-relativistic X2C level (B-TZVP in Table S1, BHandHLYP/TZVP in Figure S3) are in very good agreement with the experimental shifts both in the neat TBA solvent and in PL environments, which naturally leads to very similar TBA - PL shift difference obtained from the two-site model analysis of the experimental data.

It is clear that the DFT functional choice is more important than the choice of the basis set for atoms surrounding the Xe, as even the BHandHLYP/SVP level gives reasonable

chemical shifts for the PL. It also provides correctly signed, albeit underestimated TBA - PL shift difference due to over-/underestimated cage/TBA chemical shifts, on account of the basis set. The reason is that the DFT functional effect (DFE in Table S1) is much larger inside the cages than in the TBA solvent.

Actually, the basis set effect (BSE) is also very important as it is larger and to the opposite, *i.e.* increasing shift direction, as compared to DFE in the neat TBA solvent. This leads to an almost perfect error cancellation at the PBE/SVP level (P-SVP in Table S1) and almost correct Xe chemical shift in the neat TBA. However, the result is a clear example of the danger of reaching the right answer for the wrong reasons. This is evident as the PBE/SVP method leads to totally erroneous TBA - PL shift difference, with the wrong sign, due to the huge overestimation of the ^{129}Xe chemical shift in the **CC3**-*R* and **CC13** cages, where DFT functional and basis-set errors are to the same direction.

Therefore, the lowest but still economical computational level for NMR shielding calculations of Xe inside molecular cavities, is to carry out BHandHLYP calculations with a locally dense x2c-TZVPall-s(Xe)/x2c-SVPall(other) basis sets. Basis-set correction can be estimated either with pure a DFT GGA functional (PBE), as done currently, or with a few demanding hybrid BHandHLYP calculations for a small subgroup of samples.

2.2.1 Spin-orbit effect on the Xe shift

The effect of the relativistic spin-orbit correction (SOC) on the ^{129}Xe chemical shift was studied for a few snapshots at the zeroth-order regular approximation (ZORA)[26,27] level in the ADF[28,29] code. BHandHLYP functional with QZ4P-J/DZP basis sets for Xe/other atoms, were used[30]. Calculations including only scalar-relativistic (SR-ZORA), as well as both spin-orbit and scalar-relativistic (SO-ZORA) effects were carried out for 10 uncorrelated snapshots for all the three simulated systems. While 20 TBA solvent molecules closest to Xe atom were included in the neat TBA calculations, the 20 explicit TBA molecules surrounding the **CC3**-*R* and **CC13** cages in each snapshots were removed. This provides the pure SOC effect due to the cage only. The results are collected in Table S2. In the neat TBA, SOC is positive and only few ppm. Inside the cages, the SOC can appear with either sign and the averages are very close to zero. Due to the small SOC in all the systems, it can be neglected for the current purposes, since it is overrun by other, systematic and statistical errors.

Table S2. Spin-orbit correction (SOC) on ^{129}Xe NMR chemical shifts (δ_{Xe}) estimated at zeroth-order relativistic approximation (ZORA) level. Average (AVG) effects over 10 samples with standard deviations (STD) and standard errors of mean (SEM) are reported. See the main text for details.

	TBA solvent			**CC3-*R***			**CC13**		
Snapshot	SR[a]	SO[b]	SOC[c]	SR[a]	SO[b]	SOC[c]	SR[a]	SO[b]	SOC[c]
1	229.1	232.6	3.4	97.5	97.6	0.2	136.7	137.9	1.2
2	36.0	36.3	0.2	42.6	42.2	-0.5	1.9	1.2	-0.7
3	192.9	196.4	3.6	57.5	57.6	0.1	104.9	105.7	0.8
4	185.6	188.1	2.5	288.8	292.5	3.7	63.5	64.0	0.5
5	221.1	224.7	3.6	22.1	21.8	-0.3	-14.6	-15.7	-1.1
6	275.7	280.8	5.0	22.6	22.2	-0.3	69.8	69.6	-0.2
7	235.5	239.3	3.8	4.8	4.3	-0.5	130.4	131.3	0.9
8	145.6	149.3	3.7	76.4	76.8	0.3	67.1	67.5	0.4
9	198.1	201.6	3.5	5.6	5.1	-0.5	-10.8	-11.6	-0.8
10	195.9	199.9	4.1	-25.9	-26.9	-1.0	9.1	8.6	-0.4
AVG	191.5	194.9	**3.4**	59.2	59.3	**0.1**	55.8	55.9	**0.1**
STD	64.7	65.8	1.3	88.6	89.8	1.3	57.1	57.9	0.8
SEM	20.5	20.8	0.4	28.0	28.4	0.4	18.1	18.3	0.3

[a] Scalar-relativistic SR-ZORA result with respect to $\sigma_{\mathrm{Xe\ atom}} = 5754.5$ ppm.
[b] Spin-orbit and scalar-relativistic SO-ZORA result with respect to $\sigma_{\mathrm{Xe\ atom}} = 6647.5$ ppm.
[c] Spin-orbit correction computed as the SO-ZORA - SR-ZORA difference.

References

(1) Greenaway, R. L.; Holden, D.; Eden, E. G. B.; Stephenson, A.; Yong, C. W.; Bennison, M. J.; Hasell, T.; Briggs, M. E.; James, S. L.; Cooper, A. I. Understanding Gas Capacity, Quest Selectivity, and Diffusion in Porous Liquids. *Chem. Sci.* **2017**, *8*, 2640–2651.

(2) Kearsey, R. J.; Alston, B. M.; Briggs, M. E.; Greenaway, R. L.; Cooper, A. I. Accelerated Robotic Discovery of Type II Porous Liquids. *Chem. Sci.* **2019**, *10*, 9454–9465.

(3) Bannwarth, C.; Caldeweyher, E.; Ehlert, S.; Hansen, A.; Pracht, P.; Seibert, J.; Spicher, S.; Grimme, S. Extended Tight-Binding Quantum Chemistry Methods. *WIREs Comput. Mol. Sci.* **2020**, e01493.

(4) Bannwarth, C.; Ehlert, S.; Grimme, S. GFN2-xTB — An Accurate and Broadly Parametrized Self-Consistent Tight-Binding Quantum Chemical Method with Multipole Electrostatics and Density-Dependent Dispersion Contributions. *J. Chem. Theory Comput.* **2019**, *15*, 1652–1671.

(5) Humphrey, W.; Dalke, A.; Schulten, K. VMD: Visual Molecular Dynamics. *J. Mol. Graphics* **1996**, *14*, 33–38.

(6) Martínez, L.; Andrade, R.; Birgin, E. G.; Martínez, J. M. PACKMOL: A Package for Building Initial Configurations for Molecular Dynamics Simulations. *J. Comput. Chem.* **2009**, *30*, 2157–2164.

(7) Flyvbjerg, H.; Petersen, H. G. Error Estimates on Averages of Correlated Data. *J. Chem. Phys.* **1989**, *91*, 461–466.

(8) Frenkel, D.; Smit, B. *Understanding Molecular Simulation: From Algorithms to Applications*, 2nd ed.; Academic Press, 2002.

(9) Ahlrichs, R.; Bär, M.; Häser, M.; Horn, H.; Kölmel, C. Electronic Structure Calculations on Workstation Computers: The Program System Turbomole. *Chem. Phys. Lett.* **1989**, *162*, 165–169.

(10) TURBOMOLE V7.6 2021, a Development of University of Karlsruhe and Forschungszentrum Karlsruhe GmbH, 1989-2007, TURBOMOLE GmbH, Since 2007; Available from `http://www.turbomole.com`.

(11) Franzke, Y. J.; Weigend, F. NMR Shielding Tensors and Chemical Shifts in Scalar-Relativistic Local Exact Two-Component Theory. *J. Chem. Theory Comput.* **2019**, *15*, 1028–1043.

(12) Franzke, Y. J.; Treß, R.; Pazdera, T. M.; Weigend, F. Error-Consistent Segmented Contracted All-Electron Relativistic Basis Sets of Double- and Triple-zeta Quality for NMR Shielding Constants. *Phys. Chem. Chem. Phys.* **2019**, *21*, 16658–16664.

(13) Pollak, P.; Weigend, F. Segmented Contracted Error-Consistent Basis Sets of Double- and Triple-ζ Valence Quality for One- and Two-Component Relativistic All-Electron Calculations. *J. Chem. Theory Comput.* **2017**, *13*, 3696–3705.

(14) Perdew, J. P.; Burke, K.; Ernzerhof, M. Generalized Gradient Approximation Made Simple. *Phys. Rev. Lett.* **1996**, *77*, 3865–3868.

(15) Lantto, P.; Vaara, J. ^{129}Xe Chemical Shift by the Perturbational Relativistic Method: Xenon Fluorides. *J. Chem. Phys.* **2007**, *127*, 084312.

(16) Straka, M.; Lantto, P.; Räsänen, M.; Vaara, J. Theoretical Predictions of Nuclear Magnetic Resonance Parameters in a Novel Organo-Xenon Species: Chemical Shifts and Nuclear Quadrupole Couplings in HXeCCH. *J. Chem. Phys.* **2007**, *127*, 234314.

(17) Roukala, J.; Maldonado, A. F.; Vaara, J.; Aucar, G. A.; Lantto, P. Relativistic Effects

on Group-12 Metal Nuclear Shieldings. *Phys. Chem. Chem. Phys.* **2011**, *13*, 21016–21025.

(18) Lantto, P.; Standara, S.; Riedel, S.; Vaara, J.; Straka, M. Exploring New ^{129}Xe Chemical Shift Ranges in HXeY Compounds: Hydrogen More Relativistic Than Xenon. *Phys. Chem. Chem. Phys.* **2012**, *14*, 10944–10952.

(19) Straka, M.; Lantto, P.; Vaara, J. Toward Calculations of the ^{129}Xe Chemical Shift in Xe@C_{60} at Experimental Conditions: Relativity, Correlation, and Dynamics. *J. Phys. Chem. A* **2008**, *112*, 2658–2668.

(20) Roukala, J.; Zhu, J.; Giri, C.; Rissanen, K.; Lantto, P.; Telkki, V.-V. Encapsulation of Xenon by a Self-Assembled Fe_4L_6 Metallosupramolecular Cage. *J. Am. Chem. Soc.* **2015**, *137*, 2464–2467.

(21) Selent, M.; Nyman, J.; Roukala, J.; Ilczyszyn, M.; Oilunkaniemi, R.; Bygrave, P. J.; Laitinen, R.; Jokisaari, J.; Day, G. M.; Lantto, P. Clathrate Structure Determination by Combining Crystal Structure Prediction with Computational and Experimental ^{129}Xe NMR Spectroscopy. *Chem. Eur. J.* **2017**, *23*, 5258–5269.

(22) Komulainen, S.; Roukala, J.; Zhivonitko, V. V.; Javed, M. A.; Chen, L.; Holden, D.; Hasell, T.; Cooper, A.; Lantto, P.; Telkki, V.-V. Inside Information on Xenon Adsorption in Porous Organic Cages by NMR. *Chem. Sci.* **2017**, *8*, 5721–5727.

(23) Becke, A. D. Density-Functional Exchange-Energy Approximation with Correct Asymptotic Behavior. *Phys. Rev. A* **1988**, *38*, 3098–3100.

(24) Lee, C.; Yang, W.; Parr, R. G. Development of the Colle-Salvetti Correlation-Energy Formula into a Functional of the Electron Density. *Phys. Rev. B* **1988**, *37*, 785–789.

(25) Becke, A. D. A New Mixing of Hartree–Fock and Local Density-Functional Theories. *J. Chem. Phys.* **1993**, *98*, 1372–1377.

(26) van Lenthe, E.; Baerends, E. J.; Snijders, J. G. Relativistic Regular Two-Component Hamiltonians. *J. Chem. Phys.* **1993**, *99*, 4597–4610.

(27) van Lenthe, E.; Baerends, E. J.; Snijders, J. G. Relativistic Total Energy Using Regular Approximations. *J. Chem. Phys.* **1994**, *101*, 9783–9792.

(28) te Velde, G.; Bickelhaupt, F. M.; Baerends, E. J.; Fonseca Guerra, C.; van Gisbergen, S. J. A.; Snijders, J. G.; Ziegler, T. Chemistry with ADF. *J. Comput. Chem.* **2001**, *22*, 931–967.

(29) Baerends, E. J.; Ziegler, T.; Atkins, A. J.; Autschbach, J.; Bashford, D.; Baseggio, O.; Bérces, A.; Bickelhaupt, F. M.; Bo, C.; Boerritger, P. M. et al. ADF2019, SCM, Theoretical Chemistry, Vrije Universiteit, Amsterdam, The Netherlands, `https://www.scm.com`. 2019.

(30) van Lenthe, E.; Baerends, E. J. Optimized Slater-Type Basis Sets for the Elements 1–118. *J. Comput. Chem.* **2003**, *24*, 1142–1156.
